# Airway Closure and Expiratory Flow Limitation in Acute Respiratory Distress Syndrome

**DOI:** 10.3389/fphys.2021.815601

**Published:** 2022-01-17

**Authors:** Claude Guérin, Martin Cour, Laurent Argaud

**Affiliations:** ^1^Médecine Intensive - Réanimation Hôpital Edouard Herriot Lyon, Lyon, France; ^2^Faculté de Médecine Lyon-Est, Université de Lyon, Lyon, France; ^3^Institut Mondor de Recherches Biomédicales, INSERM-UPEC UMR 955 Team 13 - CNRS ERL 7000, Créteil, France

**Keywords:** acute respiratory distress syndrome, airway closure, COVID-19, respiratory mechanics, expiratory flow limitation, positive end-expiratory pressure

## Abstract

Acute respiratory distress syndrome (ARDS) is mostly characterized by the loss of aerated lung volume associated with an increase in lung tissue and intense and complex lung inflammation. ARDS has long been associated with the histological pattern of diffuse alveolar damage (DAD). However, DAD is not the unique pathological figure in ARDS and it can also be observed in settings other than ARDS. In the coronavirus disease 2019 (COVID-19) related ARDS, the impairment of lung microvasculature has been pointed out. The airways, and of notice the small peripheral airways, may contribute to the loss of aeration observed in ARDS. High-resolution lung imaging techniques found that in specific experimental conditions small airway closure was a reality. Furthermore, low-volume ventilator-induced lung injury, also called as atelectrauma, should involve the airways. Atelectrauma is one of the basic tenet subtending the use of positive end-expiratory pressure (PEEP) set at the ventilator in ARDS. Recent data revisited the role of airways in humans with ARDS and provided findings consistent with the expiratory flow limitation and airway closure in a substantial number of patients with ARDS. We discussed the pattern of airway opening pressure disclosed in the inspiratory volume-pressure curves in COVID-19 and in non-COVID-19 related ARDS. In addition, we discussed the functional interplay between airway opening pressure and expiratory flow limitation displayed in the flow-volume curves. We discussed the individualization of the PEEP setting based on these findings.

## Introduction

Acute respiratory distress syndrome (ARDS), a non-cardiogenic pulmonary edema with lung inflammation, loss of aeration, higher intra-pulmonary shunt, lower compliance of respiratory system, and hypoxemia, is primarily driven by pneumonia, aspiration, and extra-pulmonary sepsis (Bellani et al., 2016; Thompson et al., 2017). Before the COVID-19 pandemic, it accounted for 10% of intensive care unit (ICU) admissions and supported a 28-day median mortality rate of about 35%, and >40% in the severe ARDS category (Bellani et al., 2016). With the COVID-19 pneumonia, the number of ARDS cases exploded worldwide and the mortality remained in the same range as that of non-COVID-19 (Grasselli et al., 2020; Matthay et al., 2020). The role of airways in the pathophysiology of ARDS has largely remained unknown even though airway collapsibility in this setting was suspected many years ago (Bindslev et al., 1980; Hedenstierna and McCarthy, 1980). As discussed below, the histological involvement of airways was marginally described in ARDS. However, recent data suggest that airways may play a role in the pathophysiology of both COVID-19 and non-COVID-19 ARDS.

In this narrative review, we aimed to decipher the data subtending the implication of airways in the pathogenesis of ARDS and discuss some therapeutic approaches.

## Lung Pathology of ARDS

Post-mortem examination of 7 patients in the 12 originally described as ARDS by Ashbaugh et al. reported that the lungs were heavier than normal and disclosed capillary congestion, areas of alveolar atelectasis, interstitial and alveolar hemorrhage, and hyalines membranes (Ashbaugh et al., 1967). The pulmonary vasculature and the trachea-bronchial tree were free of obstruction (Ashbaugh et al., 1967). Then, Katzenstein et al. (1976) popularized the term of diffuse alveolar damage (DAD) that has long been tightly associated with ARDS and thought of as its pathognomonic pathological feature. DAD includes lung epithelial and endothelial injury, lung edema, hyalines membranes, and then proliferation of alveolar, interstitial, and bronchial cells (Katzenstein et al., 1976). Three distinct phases have been described during the ARDS course: the exudative phase with lung edema formation, the fibro-proliferative stage, and the fibrotic stage (Thompson et al., 2017). The transition from phase 2 to phase 3 is not predictable, and the phase 3 may evolve toward either a complete recovery or a persistence of post-aggressive fibrosis that may itself recover. Interestingly, Thille et al. were able to describe these three phases from autopsy lung examination in patients who died with ARDS in the ICU (Thille et al., 2013). Over time, it turned out, however, that DAD was not the main histopathological feature of ARDS. Libby et al. in a meta-analysis of studies reporting on the open lung biopsy in patients with ARDS found a 9% rate of DAD in the 1,205 pooled patients (Libby et al., 2014). The most frequent diagnoses of the ARDS cause after lung histology assessment were interstitial lung disease (25%) and infection (24%) (Libby et al., 2014). In a subsequent study, which included 83 patients from two ICUs with non-resolving ARDS, the rate of DAD diagnosed on lung biopsies was 58% (Guerin et al., 2015a). Three factors may contribute to a lower rate of DAD than expected: (1) part of the DAD may have been related to ventilator-induced lung injury and has decreased over time with the wider use of lung-protective ventilation and lower tidal volume (Acute Respiratory Distress Syndrome Network, 2000), (2) patients with an underlying lung disease may present with the clinical ARDS figure (Guerin et al., 2015b; Aublanc et al., 2017), and (3) DAD is more frequently observed in non-resolving or fatal ARDS than in the other cases.

In patients with COVID-19, lung histopathology is close to that pertaining to classic ARDS. A meta-analysis on 27 studies providing the results of surgical lung biopsy or post-mortem lung autopsy in 195 patients who died from COVID-19, found DAD in 80% of the studies and heterogeneous histopathology (Pannone et al., 2021). The severity of lung histopathology may explain out-of-hospital cardiac arrest (Fanton et al., 2021). Copin et al. described the pattern of organizing pneumonia in 6 patients, associated with fibrin deposition in the bronchioles (Copin et al., 2020), and Fox et al. emphasized on lung microangiopathy in African-American subjects (Fox et al., 2020).

## Airway Closure in Normal Human Physiology

**Figure 1:**
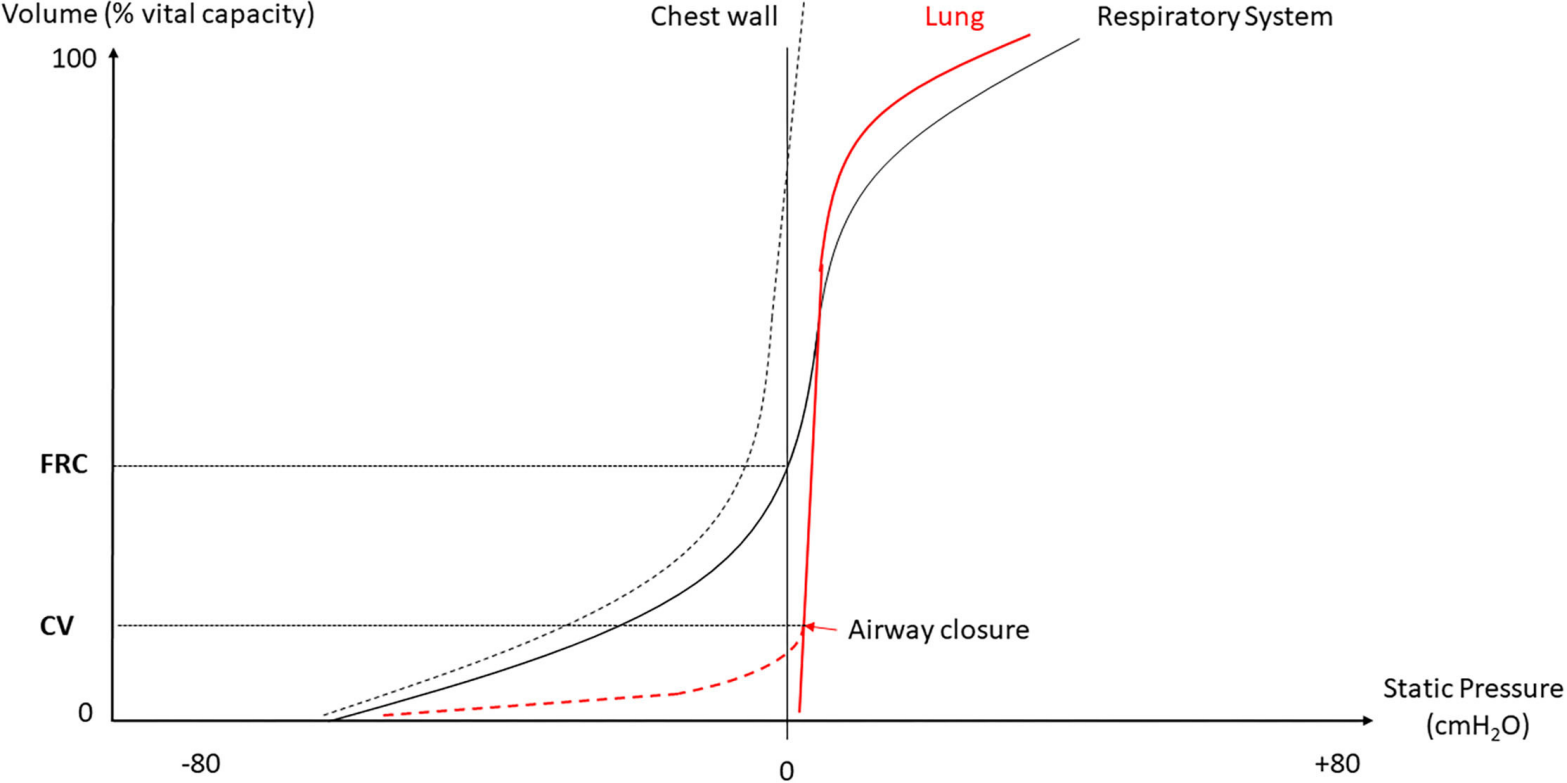
Deflation static volume-pressure (VP) curve of the lung, chest wall and whole respiratory system. FRC, functional residual capacity; CV, closing volume.

**Figure 2:**
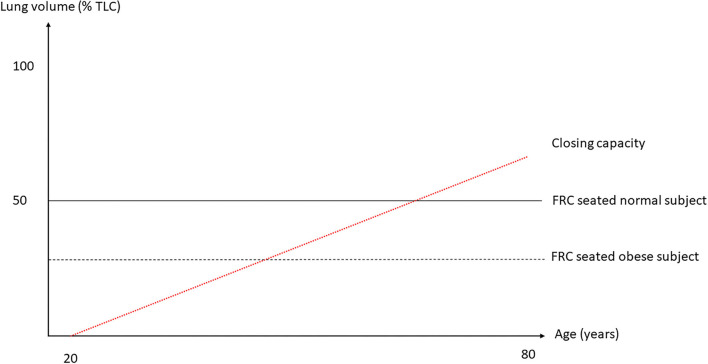
Lung volumes as a function of total lung capacity (TLC) against age. The two horizontal broken lines are FRC seated in normal and obese subject and the red broken line the trend of closing capacity with age. As closing capacity increases with age, an obese subject, in whom FRC is lower, would be likely to exhibit airway closure at a younger age than a non-obese subject. Not shown here is the fact that FRC goes down with increasing body mass index.

In normal humans, the lung deflates from total lung capacity (TLC) down to 10% TLC due to its own elastic recoil. At zero trans-pulmonary pressure (P_L_), the airways may be kept open under the action of their internal structure and of the traction of the surrounding lung parenchyma that stems from the lung elastic recoil. The cartilaginous walls of the central airways make them more likely to stay open while the patency of non-cartilaginous peripheral airways depends on the radial traction of the surrounding lung. At low lung volume, the elastic recoil is less and so the radial traction is also less and hence the peripheral airways are more likely to collapse. The pattern of deflation in a static volume-pressure (VP) curve of a normal subject in the sitting position is different in the absence or presence of airway closure (Figure 1). Though the lung does not fully empty over the vital capacity range in absence of airway closure (Agostoni and Mead, 1964; Agostoni and Hyatt, 1986), the presence of airway closure, which happens below functional residual capacity (FRC), deviates the static lung VP curve to the left (Sutherland et al., 1968) (Figure 1). With the use of the VP curve method, different values of critical P_L_ at which airways start closing have been found across animal species and experimental preparations. When airways start closing at 4 cm H_2_O P_L_ in excised lungs dogs (Glaister et al., 1973), they were found still open at negative P_L_ in *in situ* closed-chest lungs rabbits (Cavagna et al., 1967). In closed-chest pigs, cats, dogs, and rabbits, the airways remain open at P_L_ of −8.3 (Bayle et al., 2006), −12.4 (Cavagna et al., 1967), −11.9 (Cavagna et al., 1967), and −12.7 (Cavagna et al., 1967) cmH_2_O, respectively. The closing volume is the lung volume at which airways start closing and the closing capacity is the sum of closing volume and residual volume. Both can be measured by the single breath N_2_ washout after 100% oxygen inhalation as a distinct phase IV (McCarthy et al., 1972). The closing capacity increases with age and FRC is lower in obese than in non-obese subjects (Figure 2). Therefore, airway closure is more likely to occur at a younger age and in obese than in non-obese patients (Figure 2).

When the airways of mammalians are closed, the lungs still contain some air behind the closure in the amount of roughly 0.5 ml/100 g lung tissue (Greaves et al., 1986), meaning that alveoli closed after the airways became closed. In a very elegant *in vivo* experiment by Cavagna et al. in animals (Cavagna et al., 1967), deflation lung VP curves were drawn during: (1) absorption of pure oxygen after tracheal clamping and (2) withdrawal of air-filled lungs from the trachea. The comparison of VP curves in both experimental conditions showed that terminal lung units remained in continuity with the trachea till a negative P_L_ in order of −2 cm H_2_O happens, meaning that both airways and alveoli have an elastic resistance to collapse. The mechanisms of that resistance to collapse may result from the erectile effect of vascular distension at low lung volume that tethered the peripheral airways (Goldberg et al., 1975). The sequence of closure, airways then alveoli, allows gas exchange to continue behind the closure. Once the alveoli are closed, atelectasis occurs and reopening atelectatic lung requires greater P_L_ than that required for reopening closed airways.

When closing capacity becomes near to FRC (Figure 2), the likelihood of tidal expiratory flow limitation (EFL) increases with the ease of airways to get closed. In normal subjects, any increase in expiratory driving pressure (alveolar pressure minus atmospheric pressure) is followed by an increase in expiratory flow for a given lung volume. EFL occurs whenever the expiratory flow does not increase after an increase in expiratory driving pressure. This feature is the expression of airway collapse that occurs when the intraluminal pressure of the airways is lower than the external pressure, which is the pleural pressure. Tidal EFL heralds airway closure. Its measurement can be done by the atmospheric method that consists in changing abruptly the airway-to-atmospheric pressure gradient over one breath.

In normal subjects under mechanical ventilation and general anesthesia, airway closure was measured at 4.5 cm H_2_O P_L_ in some studies (Hedenstierna and McCarthy, 1980) and atelectasis was disclosed by using CT scan (Gunnarsson et al., 1989). Both airway closure and atelectasis contributed to gas exchange abnormalities that occurred early after anesthesia induction in normal subjects (Rothen et al., 1998).

## Airway closure in ARDS

### Causes

In patients with ARDS many factors can contribute to airway closure:

1. Intraluminal factors:
  a. The surfactant impairment in qualitative or quantitative terms will reduce the surface tension at the air-liquid interface in the terminal bronchioles and favor closure (Albert, 2012; Coudroy et al., 2019).
  b. Some fluid may accumulate in the lumen of the airways and in the alveoli, forming a foam that may completely or partly obstruct the small airways lumen (Wilson et al., 2001).
  c. Absorption atelectasis in the terminal lung units of lung regions with low ventilation-to-perfusion ratio can result from higher levels of F_I_O_2_ (Aboab et al., 2006).

2. Parietal factors:
  a. Some bronchoconstriction may arise from the mediators that are released during the acute inflammatory process (D'Angelo et al., 2008).

3. External factors:
  a. The loss of elastic recoil that results from elastic fibers destruction will reduce the tethering effect of the radial traction of the surrounding lung parenchyma.
  b. The airways may be compressed by the higher mass of the lung according to the sponge model of ARDS (Gattinoni et al., 2013).

### Consequences

In turn, the reduction of airways lumen will increase the airway flow resistance. Indeed, an increased airway flow resistance has been described in ARDS (Wright and Bernard, 1989; Eissa et al., 1991). However, this finding was related to the reduction in aerated lung volume (Pelosi and Rocco, 2007). Functionally speaking, ARDS is a restrictive lung disease with a reduction in lung volumes. The FRC is lower than the expected normal values and the reduction in FRC goes up with the increased ARDS severity (Cressoni et al., 2015). The baby lung important concept originated from this finding (Gattinoni and Pesenti, 2005).

Another consequence of airway closure is that it would favor the repeated opening and closure of the terminal respiratory units from breath to breath that would further injure the lung. This low volume barotrauma is another mechanism of ventilator-induced lung injury (Muscedere et al., 1994). When it occurs in those lung regions near to those consolidated and not re-openable, considerable forces are applied that produce major lung stress (Mead et al., 1970).

What is totally unknown in ARDS is the role of collateral ventilation that should theoretically prevent some alveolar collapse by feeding with air through the Köhn pores step by step the neighborhood airways tree (Woolcock and Macklem, 1971; Hogg et al., 1972).

### Expiratory Flow Limitation and Airway Closure

**Figure 3:**
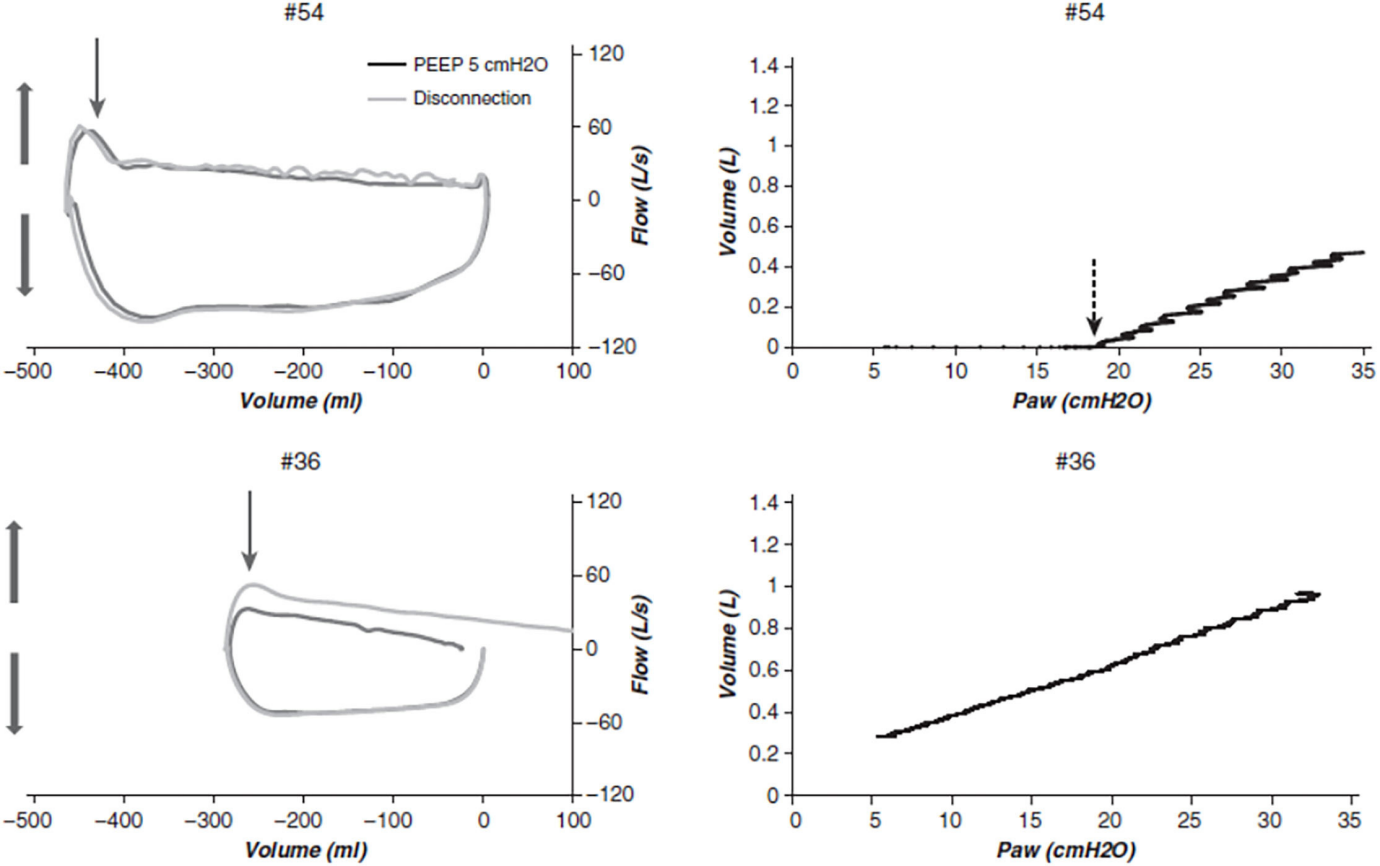
Confrontation of flow-volume and VP relationships in two patients with acute respiratory distress syndrome (ARDS). Flow–volume and pressure–volume (PV) curves from two representative patients (#54 and #36) in the present study. **Left**: flow–volume loops during baseline mechanical ventilation and after disconnecting (thin arrows) to the atmosphere. **Upper**: A patient with expiratory flow limitation (EFL) over the whole expiratory volume, meaning that the whole breath happens in the closing volume. **Lower**: A patient without EFL. Thick vertical arrows indicate expiration (upward) and inspiration (downward) directions. The expiratory time constant was measured during disconnection as the time required to exhale 63% of the insufflated volume. **Right**: Corresponding low-flow PV curves. Upper: An early increase in pressure does not result in an increase in volume up to a point (vertical dashed arrow), at which the volume suddenly increases. This pattern is consistent with airway reopening in this patient with EFL. The airway opening pressure was determined by visual inspection, and the compliance of the PV curve from PEEP with the Paw was computed. **Lower**: A sustained increase in volume from the onset of pressurization indicates the absence of a critical airway opening pressure in this patient without EFL. Paw, airway pressure; PEEP, positive end-expiratory pressure. Reprinted with permission of the American Thoracic Society. Copyright © 2021 American Thoracic Society. All rights reserved. Cite: Yonis, Mortaza, Baboi, Mercat, Guérin/2018/Expiratory Flow Assessment in patients with ARDS. A reappraisal/AJRCCM/198/Pages 131-134. The American Journal of Respiratory and Critical Care Medicine is an official journal of the American Thoracic Society.

As mentioned above, tidal EFL and airway closure are distinct phenomena and their temporal occurrence is not fully understood. However, both have been described in patients with ARDS. In a very elegant study using the atmospheric method (Valta et al., 1994), tidal EFL was found in 8 out of 10 patients with ARDS under zero end-expiratory pressure (Koutsoukou et al., 2000). In a subsequent study, the same authors found that tidal EFL was present in 7 patients out of 13 on zero end-expiratory pressure and was no longer present at positive end-expiratory pressure (PEEP) 5 cm H_2_O in 2 patients and 3 others at PEEP 10 cm H_2_O (Armaganidis et al., 2000). It is not surprising that some patients became not flow limited on PEEP if the latter is above the critical pressure at which those airways would collapse. These early pioneering studies were done when the principles of lung-protective mechanical ventilation were not widely applied. Furthermore, a PEEP of at least 5 cm H_2_O is now mandatory to define ARDS according to the Berlin definition (Ranieri et al., 2012); this minimal PEEP has to be set at the ventilator unless the upper safety limit of plateau pressure is surpassed (a condition, which occurs in late ARDS with a fibrotic lung or a very low baby lung). In the current era of lung-protective ventilation, Yonis et al. found that tidal EFL measured with the atmospheric method was present in 13 out of 65 patients with ARDS under a PEEP of 5 cm H_2_O in semi-recumbent position (Figure 3). Patients with tidal EFL had higher body mass index, higher total PEEP, and higher ICU mortality than patients without tidal EFL for similar ventilator settings (Yonis et al., 2018). In a subsequent study on 25 patients with ARDS enrolled in two centers, tidal EFL measured with the atmospheric method was observed in 8 of them (Guerin et al., 2020). Patients with tidal EFL had higher lung elastance than those without EFL (Guerin et al., 2020). It should be noted that in experimental porcine models of ARDS (saline lavage with surfactant depletion and oleic acid injection), tidal EFL was not disclosed on zero end-expiratory pressure, casting some doubts about the relevance of experimental models in their extrapolation to human ARDS (Guérin et al., 2008).

Some fresh air has recently blown up onto the inspiratory VP curves. Chen et al. showed in patients with ARDS during the low inflation of the respiratory system that in some of them, the lung volume did not change until a certain airway pressure was reached and above which the volume suddenly increased linearly with further pressure (Chen et al., 2018). A pattern like this (Figure 3) suggests a critical opening pressure, which was henceforth called airway opening pressure (AOP). To make this finding more robust, the authors measured the compliance of the ventilator circuit and found that the slope of the VP relationship from the onset of inflation to AOP was equal to that of the compliance of the circuit. Accordingly, from onset of inflation to AOP air circulation happens within the ventilator circuit only. It turned out that AOP was found in almost 30% of either classic ARDS or COVID-19-related ARDS (Table 1). The prevalence of AOP was even greater in obese, which is not surprising as obesity increased the elastic load superimposed to the chest wall (Coudroy et al., 2020). The static elastic chest wall compliance is normal in obese, i.e., the chest wall is not stiffer, it is overloaded (Behazin et al., 2010).

**Table 1:** Values of airway opening pressure in non-COVID-19 or COVID-19-related acute respiratory distress syndrome.

**References**	**Cause of ARDS**	**Percentage of patients with AOP (N/total)**	**Median or mean AOP (cmH_**2**_O)**
Chen et al. (2018)	Non-COVID-19	27 (8/30)	13
Yonis et al. (2018)	Non-COVID-19	32 (21/65)	NA
Maggiore et al. (2001)	Non-COVID-19	33 (15/45)	5–20
Guerin et al. (2020)	Non-COVID-19	52 (13/25)	9
Haudebourg et al. (2020)	Non-COVID-19	10 (3/30)	5
Grieco et al. (2020)	Non-COVID-19	30 (10/30)	NA
Haudebourg et al. (2020)	COVID-19	40 (12/30)	8
Stevic et al. (2021)	COVID-19	33 (8/24)	NA
Cour et al. (2021)	COVID-19	22 (4/18)	NA
Beloncle et al. (2020)	COVID-19	24 (6/25)	8
Grieco et al. (2020)	COVID-19	7 (2/30)	NA
Pan et al. (2020)	COVID-19	0 (0/12)	NA
Brault et al. (2020)	COVID-19	44 (12/27)	8

*ARDS, acute respiratory distress syndrome; AOP, airway opening pressure; NA, not available*.

Simultaneous assessment of EFL and AOP in patients with ARDS at PEEP 5 in semi-recumbent position found that both are not synonymous (Guerin et al., 2020). EFL was observed in 8 patients, 7 of them exhibiting AOP, and AOP was present in 13 patients, meaning that 6 patients had AOP without EFL. Patients with AOP had higher lung dynamic elastance and higher mechanical power than patients without AOP and the same was true between patients with EFL and without EFL. The additional lung tissue resistance measured with the airway occlusion technique at the end of inspiration was higher in patients with EFL than without EFL but did not differ between patients with and without AOP, and the interrupter resistance, i.e., the resistance in the conducting airways did not differ between patients with EFL and without EFL. This may suggest that EFL occurs in the most distal parts of the small airways. AOP was also present in patients with COVID-19 related ARDS as mentioned above but EFL was not explored in them.

X-ray techniques in the Synchrotron facilities can dynamically image the lung *in vivo* in three dimensions at a resolution of 20 μm. Thanks to this technique, some light was shed on airway closure. In rabbits, small airways closure occurred more frequently when the lungs had been injured than in the animals whose lungs were normal. Furthermore, airway closure can occur at more than one site in a given small airway during mechanical ventilation (Broche et al., 2019). Moreover, small airway closure was observed either during inspiration or during expiration in different lung areas (Fardin, 2019). One limitation of this technique is that the animals received mechanical ventilation in the erect position rather than the supine or prone position.

## Therapeutic Aspects

One pharmacological approach and one ventilator setting will be discussed in this section as examples of therapeutic implications of the previous considerations.

### Bronchodilator Agents

Beta-2 adrenergic receptors agonists relax the smooth muscle fiber within the airway wall of the cartilaginous airways in case of bronchoconstriction and hence increase their lumen and reduce airway resistance. If airway closure and/or EFL mostly result from bronchoconstriction, its relief should make the airways larger. Wright et al. found that aerosolized metaproterenol as compared with placebo significantly reduced air-flow resistance and increased dynamic lung compliance in 8 patients with ARDS intubated and mechanically ventilated (Wright and Bernard, 1989). Pesenti et al. found that intravenous salbutamol reduced air-flow resistance but did not change additional tissue resistance in patients with ARDS (Pesenti et al., 1993). Koutsoukou et al. found that nebulized salbutamol did not change the amount of EFL in patients with ARDS (Koutsoukou et al., 2000), a finding that is in accordance with the previous results of Guérin et al. (lung tissue resistance higher in EFL patients) (Guerin et al., 2020) and Pesenti et al. (no effect of salbutamol on lung tissue resistance) (Pesenti et al., 1993). Beta-2 adrenergic receptors agonists have not only bronchodilatating properties in broncho-constricted airways but also contribute to lung edema clearance. This results from the upregulation of the apical Na/K ATPase in type II alveolar cells. From this basis, Perkins et al. found that intravenous salbutamol significantly reduced the extravascular lung water as compared with a placebo group (Perkins et al., 2006) and improved epithelial repair (Perkins et al., 2008) in patients with ARDS. However, random controlled trials (RCTs) did not confirm these physiological benefits. One trial was stopped early for the safety concern of higher mortality in the salbutamol group (Gao Smith et al., 2012). In another trial, aerosolized albuterol did not change the patient outcome as compared with placebo (Matthay et al., 2011). Therefore, the use of beta-2 adrenergic receptors agonists is not recommended in patients with ARDS.

### Setting PEEP

**Figure 4:**
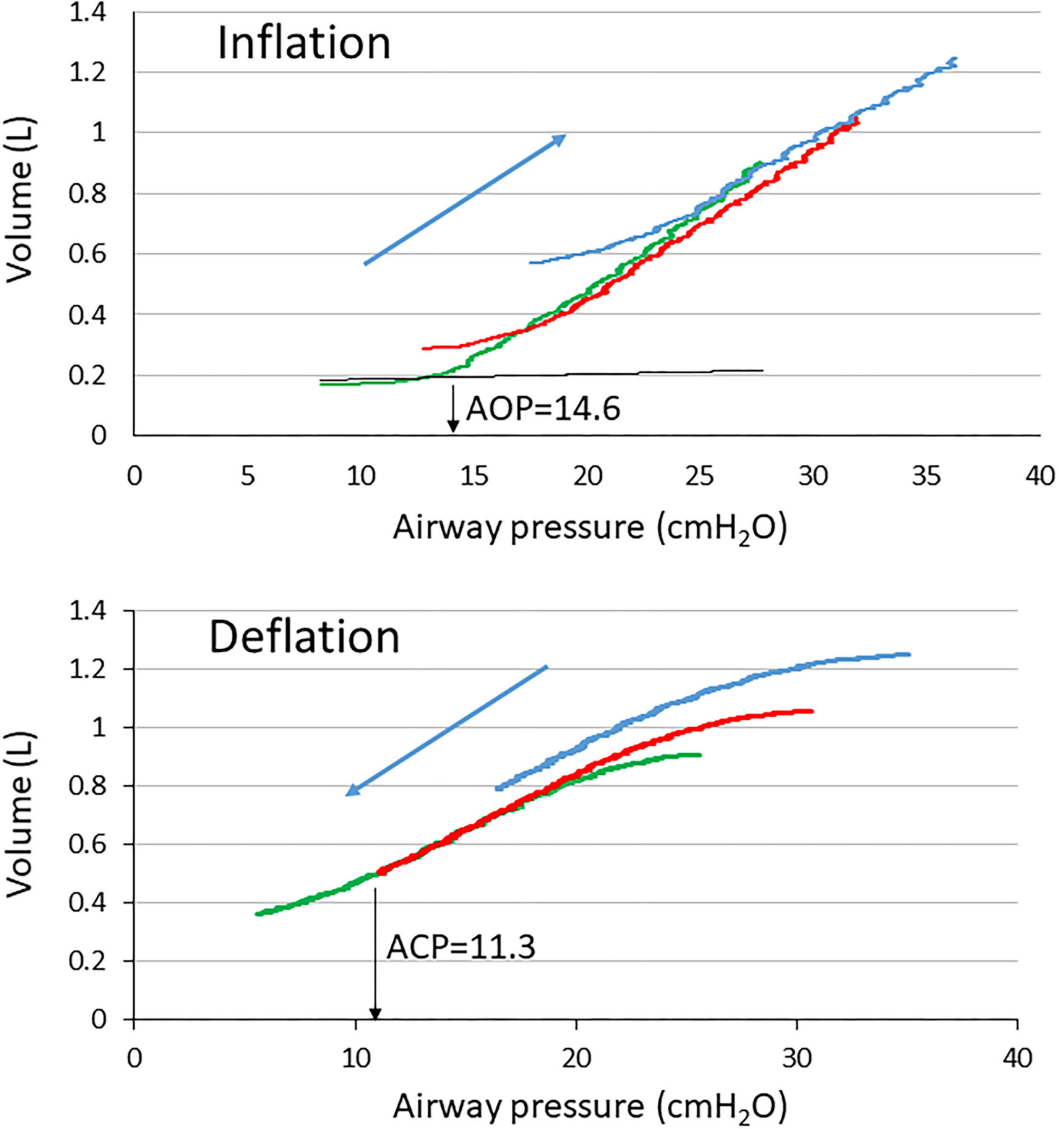
Volume-Pressure curves of the respiratory system at PEEP of 5 (green), 10 (red), and 15 (blue) cmH_2_O in a patient with ARDS with EFL. The black broken curve is the curve of the ventilator tubing used to define airway opening pressure (down black arrow AOP) at PEEP 5. ACP, airway closing pressure (down black arrow ACP).

Positive end-expiratory pressure is an expiratory ventilator setting that allows to maintain the lung recruitment generated during the preceding inspiration or resulting from a voluntary recruitment maneuver. During the tidal breathing setting, PEEP should therefore be selected with the goal to minimize the tidal recruitment/derecruitment, i.e., atelectrauma. However, atelectrauma is linked to recruitability of the lung. In ARDS patients with a high potential of recruitment, the risk of atelectrauma is higher than in those with a lower recruitability at low PEEP (Caironi et al., 2009). Setting PEEP based on the presence of airway closure indicators is therefore attractive. As discussed above, the presence of AOP may reflect airway closure and hence be used to set PEEP. At this point, some considerations should be taken into account. Hickling provided a comprehensive mathematical model of the series of events that occurred during incremental PEEP starting from a totally degassed lung up to 50 cm H_2_O plateau pressure followed by decremental PEEP from this fully recruited lung to zero volume (Hickling, 2001). In this model, open-lung PEEP was defined as the PEEP that maintained aerated 97.5% of the alveoli in the most dependent parts of the lungs. The open-lung PEEP was made dependent on both the superimposed pressure due to gravity (0 cm H_2_O in the non-dependent and 14.5 cm H_2_O in the dependent lung) and the critical closing pressure set in the 0–4 cm H_2_O range. The critical opening pressure was the P_L_ above which alveoli suddenly increased volume. Given a 18.5 cm H_2_O set open-lung PEEP and a 0–20 cm H_2_O range of critical opening pressure, the PEEP level needed to maximize the compliance was 19 cm H_2_O during incremental limb and 16 cm H_2_O during decremental PEEP, and 20 and 16 cm H_2_O, respectively, for a 2 cm H_2_O critical closing pressure (Hickling, 2001). Therefore, based on the computation of compliance, the open-lung PEEP is lower during deflation than inflation. It has been shown that the lung recruitment continued well above the “knee” (Gattinoni et al., 1987) or lower inflection point on the inflation VP curve (Crotti et al., 2001; Pelosi et al., 2001) and was a continuous process during insufflation, so that the point at which on VP curve the compliance started to decline would rather identify the end of recruitment (Jonson et al., 1999). However, it is likely that setting PEEP below this “knee” would be harmful to the lung as it should not prevent atelectrauma (Downie et al., 2004). Indeed, the “knee” correlates with the lower critical opening pressure (Hickling, 1998). One could argue that setting PEEP above AOP, being an opening pressure, makes sense if it is also a closing pressure, i.e., a pressure at which airways start closing. Due to the lung hysteresis, the closing pressure is different (e.g., lower) from the opening pressure. However, the identification of such a critical closing pressure was not so clear in ARDS and the “knee” was not an indication of airway closure when using VP curves at different PEEP in patients with ARDS (Maggiore et al., 2001). When performing slow deflation from zero end-expiratory pressure at constant flow up to a complete closure in an experimental model of ARDS, we found that airways remained open over a substantial range of airway pressure (Bayle et al., 2006), which is in line with the fact that EFL was not disclosed in this kind of experimental setting. However, since AOP happens and assuming it reflects airway reopening, airway closure should have occurred during the preceding expiration. In the study on 25 patients with ARDS already mentioned, deflation VP curves at constant low flow were performed (Guerin et al., 2020). In the patient shown in Figure 4, who had EFL at PEEP 5 cm H_2_O, the AOP was 14.6 cm H_2_O and increased with increasing PEEP, indicating that the closure was not overwhelmed up to PEEP 15, and indeed EFL was still present at that PEEP. On the deflation VP curve, the closing pressure at PEEP 5 disclosed from an unbiased analysis was lower than AOP (Figure 4).

In conclusion, airway closure happens in patients with ARDS, but the location within the airway tree and the mechanisms which originate in, need further investigation. Combining AOP and EFL assessment may help better define the pattern of airway closure and help better PEEP selection. Assuming that EFL informs about small airway collapse, for a given AOP, PEEP would be more likely to reopen the airways and maintain lung volume in the presence than in the absence of EFL.

## Author Contributions

CG drafted the first version of the manuscript. MC made substantial revisions. LA made substantial revisions. All authors contributed to manuscript revision, read, and approved the submitted version.

## Conflict of Interest

The authors declare that the research was conducted in the absence of any commercial or financial relationships that could be construed as a potential conflict of interest.

## Publisher's Note

All claims expressed in this article are solely those of the authors and do not necessarily represent those of their affiliated organizations, or those of the publisher, the editors and the reviewers. Any product that may be evaluated in this article, or claim that may be made by its manufacturer, is not guaranteed or endorsed by the publisher.
